# The Impact of Dietary Energy Intake Early in Life on the Colonic Microbiota of Adult Mice

**DOI:** 10.1038/srep19083

**Published:** 2016-01-08

**Authors:** Jinyu Xu, Jeffrey D. Galley, Michael T. Bailey, Jennifer M. Thomas-Ahner, Steven K. Clinton, Susan E. Olivo-Marston

**Affiliations:** 1 The Ohio State University Ph.D. Program in Nutrition, The Ohio State University, Columbus, OH 43210, USA; 2 Division of Epidemiology, College of Public Health, The Ohio State University, Columbus, OH 43210, USA; 3 Division of Biosciences, College of Dentistry, The Ohio State University, Columbus, OH 43210, USA; 4 Comprehensive Cancer Center, The Ohio State University, Columbus, OH 43210, USA; 5 Institute for Behavioral Medicine Research, The Ohio State University Wexner Medical Center, Columbus, OH 43210, USA; 6 Center for Microbial Pathogenesis, The Research Institute at Nationwide Children’s Hospital, Columbus OH 43205, USA; 7 Division of Medical Oncology, Department of Internal Medicine, The Ohio State University, Columbus, OH 43210, USA

## Abstract

The complex and dynamic interactions between diet, gut microbiota (GM) structure and function, and colon carcinogenesis are only beginning to be elucidated. We examined the colonic microbiota and aberrant crypt foci (ACF) in C57BL/6N female mice fed various dietary interventions (control, energy restricted and high-fat) provided during two phases (initiation and progression) of azoxymethane (AOM)-induced early colon carcinogenesis. During progression (wks. 22–60), a high-fat diet enhanced ACF formation compared to a control or energy restricted diet. In contrast, energy restriction during initiation phase (wks. 3–21) enhanced ACF burden at 60 weeks, regardless of the diet in progression phase. Alterations in GM structure during the initiation phase diet were partially maintained after changing diets during the progression phase. However, diet during the progression phase had major effects on the mucosal GM. Energy restriction in the progression phase increased Firmicutes and reduced Bacteroidetes compared to a high-fat diet, regardless of initiation phase diet, suggesting that diet may have both transient effects as well as a lasting impact on GM composition. Integration of early life and adult dietary impacts on the colonic microbial structure and function with host molecular processes involved in colon carcinogenesis will be key to defining preventive strategies.

The commensal microbiota of the colon are hypothesized to be major determinants of health and disease, including chronic inflammatory syndromes and colon carcinogenesis^1^. Recent advances in high throughput genomic analysis of the colonic microbiota are leading to the emergence of several key concepts. At any given time, the intestinal microbiota comprises over 100,000 billion bacterial cells^2^. The colon microbiota is defined by a complex and dynamic interplay between host genetic variables and multiple environmental factors, including foods and nutrients^3^. In the developing infant, maternal inoculation at birth and breastfeeding impact microbiota composition. After the first three years of life, the general structure of the microbiota is relatively consistent throughout healthy adult life^4^,^5^, but emerging data demonstrates aging, host physiology and health, pharmaceutical agents, and diet as important factors impacting variation in the microbiota structure and function^3^. Transient shifts on the gut microbiota (GM) have been observed following dietary interventions^6^ and more persistent changes followed antibiotic administration^7^. It is likely that the responses of microbiota to a specific intervention depend upon not only the type and severity of the intervention but also the present community structure.

A western dietary pattern coupled with a sedentary lifestyle, characterized by a diet rich in fat and calories, is one of the strongest risk factors for obesity and may contribute to a greater incidence of colon cancer^8^,^9^. Indeed, diets mimicking characteristics of the affluent dietary pattern have been modeled in experimental colon carcinogenesis and demonstrated cancer promoting activity^10^,^11^. A high-fat dietary pattern leading to obesity in rodent models is associated with changes in the microbiota^6^,^12^,^13^. Yet, the impact on the microbiota and the pathogenesis of colon carcinogenesis has been studied in only a few reports with heterogeneous results^6^,^12^,^13^,^14^,^15^. Conversely, energy restriction in experimental models reduces adiposity and inhibits experimental colon carcinogenesis^16^. The possibility that energy restriction may impact colonic health and cancer risk via changes in the structure or function of microbiota is also under study^17^,^18^. The complexity regarding how the microbiota impacts colon cancer risk is best illustrated by the findings that commensal bacteria have shown a detrimental effect on colon tumorigenesis in spontaneous colitis model^19^,^20^, but a beneficial role in a chemically-induced colon cancer model^21^,^22^.

The impact of specific dietary changes over the life cycle on the colonic microbiota as well as its potential association with colon carcinogenesis is uncertain. Evidence is accumulating that the diets introduced to children at the time of weaning may influence microbial diversity in later life^23^, however, the long-term impacts of diet during early life stages on gut microbiota at later time points in life are not well understood. In the present study, we examine the impact of energy balance, both diet restriction and an obesity-inducing high-fat diet, during the initiation (wks. 3–21) and progression phases (wks. 22–60) of AOM-induced colon carcinogenesis. In parallel, we document the changes in the adult microbiome at the end of the study that are impact by early dietary patterns and the subsequent change in energy balance. These studies may help investigators establish new hypotheses regarding the host and microbial interactions involved in colon carcinogenesis.

## Results

### Body measurements altered by diet

**Figure 1:**
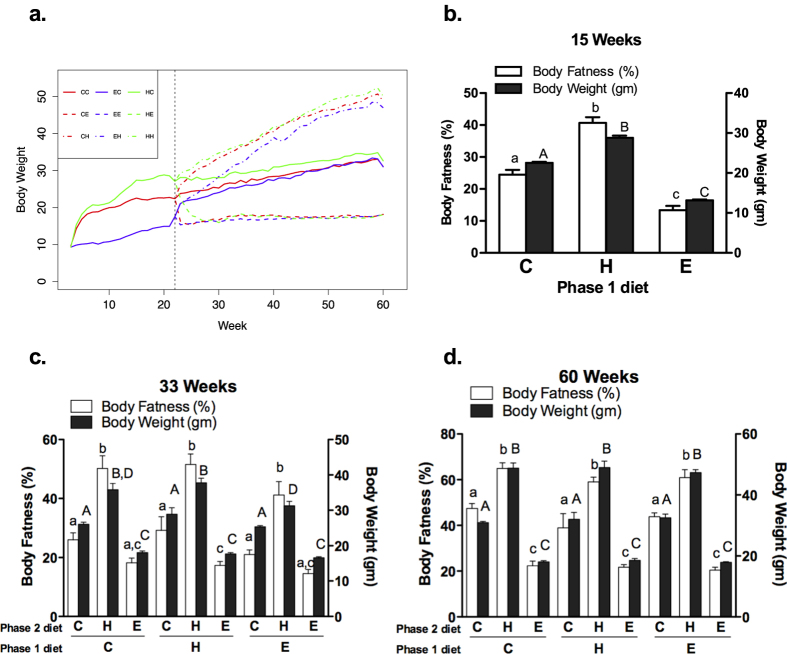
Body weights and body fatness over time. Average body weight (gm) of each diet group from 3 to 60 weeks of age (**a**). C57BL/6N female mice were weaned at 3 weeks of age and randomly assigned to control, high-fat, or energy restriction diet until 21 weeks of age (phase 1). From 22 to 60 weeks of age (phase 2), animals either remained on the same diet or switched to one of the other two diets they were not fed in phase 1, by which the study was expanded to 9 arms. C-C: control-control; C-E: control-energy restriction; C-H: control-high fat; E-C: energy restriction-control; E-E: energy restriction- energy restriction; E-H: energy restriction-high fat; H-C: high fat-control; H-E: high fat- energy restriction; H-H: high fat-high fat. Phase 1 diets were presented by different colors with red for CON, green for HF, and blue for ER. Phase 2 diet were presented by different line formats with solid line for CON, dot-dash line for HF, and dash line for ER. Body fat (clear bar) and body weights (dark bar) of a subset of animals from at 15 (b, n = 9), 33 (c, n = 4 ~ 6), and 60 (d, n = 4 ~ 6) weeks were shown between diet groups (n = 4 ~ 9). Different letters in upper case denote significant differences in body weight. Different letters in lower case denote significant differences in body fatness. p < 0.05; one-way ANOVA and Bonferroni adjustment. Data are means ± SEM.

Body weights (means ± SEM) of mice were significantly impacted by dietary intervention (Fig. 1a). At 21 weeks of age (end of initiation phase), mice fed CON (n = 81), HF (n = 43), and ER (n = 62) diets weighed 22.71 ± 0.17 gm, 28.57 ± 0.76 gm, and 14.89 ± 0.12 gm, respectively (p < 0.0001). At the end point (60 weeks of age), mice on the HF diet during the progression phase had higher body weights than mice on CON or ER, regardless the diet they were fed during initiation (p < 0.0001). The average body weight for the nine groups were as follows: H-H (n = 13): 50.11 ± 1.48 gm; C-H (n = 27): 48.80 ± 1.45 gm; E-H (n = 18): 46.97 ± 2.03 gm; H-C (n = 17): 32.62 ± 1.22 gm; C-C (n = 27): 31.06 ± 0.68 gm; E-C (n = 20): 31.00 ± 0.85 gm; H-E (n = 13): 18.06 ± 0.55 gm; C-E (n = 27): 18.14 ± 0.31 gm; and E-E (n = 24): 18.06 ± 0.20 gm (from top to bottom in Fig. 1a).

Glucose tolerance testing was impacted by dietary patterns and showed a strong association with changes in body weight (Supplementary Figure S1). The diet-induced differences in body fatness, as defined by imaging, likewise paralleled the body weights at each time point. MRI images of body fat for representative mice have been presented in supplementary materials (Supplementary Figure S2). At 15 weeks of age, mice on the high-fat diet had 40.7% body fat, compared to 24.5% in control mice, and 13.3% in those on the ER diet. (Fig. 1b, p < 0.05). At 33 weeks, the diets during the progression phase significantly influenced body fatness, regardless of the diets consumed during initiation (Fig. 1c). However, among mice on ER diet during the progression phase, mice from the ER diet group in initiation continued to show a lower percentage of fat than mice fed the CON or HF diet group during initiation (E-E = 14.5%, C-E = 18.2%, and H-E = 17.3%, p < 0.05). At 60 weeks, the impacts of the diet from the initiation phase on body fatness vanished and a significant difference only existed based-upon the diets fed during the progression phase (Fig. 1d).

### Dietary impacts on colonic ACF

**Figure 2:**
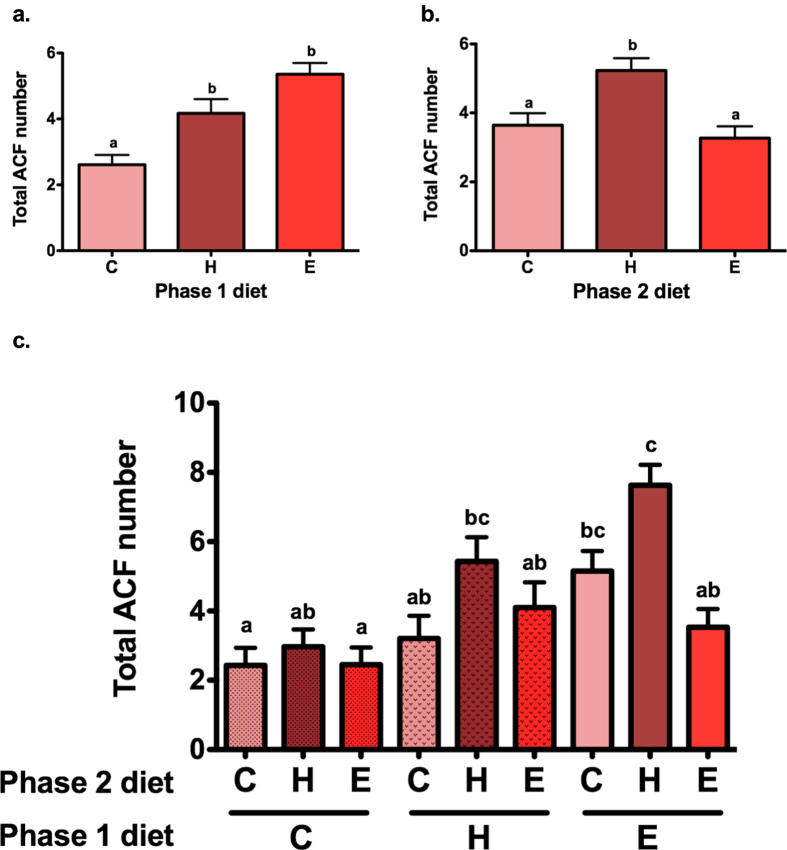
Dietary impacts on colonic ACF. Dietary patterns in phase 1 and phase 2 altered total colonic ACF numbers at the end of the study (60 weeks) (**a,b**). HF diet and ER diet in phase 1 (before and during colon carcinogenesis initiation) enhanced ACF numbers at the end of the study (**a**). In phase 2 (progression phase of carcinogenesis), HF diet increased ACF burden at the end of the study, compared to CON and ER diet (**b**). Pair-wise comparisons among nine combinations of dietary patterns in the lifespan showed similar trends (**c**), with mice in E-H (ER-HF) group showing the most burden of ACF compared to other groups. Different letters denote significant differences in total number of ACF. p < 0.05; two-way ANOVA and Bonferroni adjustment. Data are means ± SEM.

Given the unexpected observation that six weekly injections of AOM only induced tumors/polyps in six animals out of 186, we lacked sufficient power to examine the impact of dietary combinations on tumor formation. However, the number of aberrant crypt foci (ACF), a precancerous lesion, was carefully examined in segments of the proximal, medial and distal colon (Fig. 2). Mice provided the ER diet during initiation had significantly higher ACF number than those on the CON or HF diet during the same period (Fig. 2a). Mice on the ER diet during progression, however, had significantly less ACF number than those on CON diet during progression, while those on the HF diet during the same time period had the most ACF (Fig. 2b). In pair-wise comparisons among all nine groups, mice in E-H group (ER-HF) had the most ACFs compared to other groups (Fig. 2c).

### Dietary impacts on colonic microbiota

**Figure 3:**
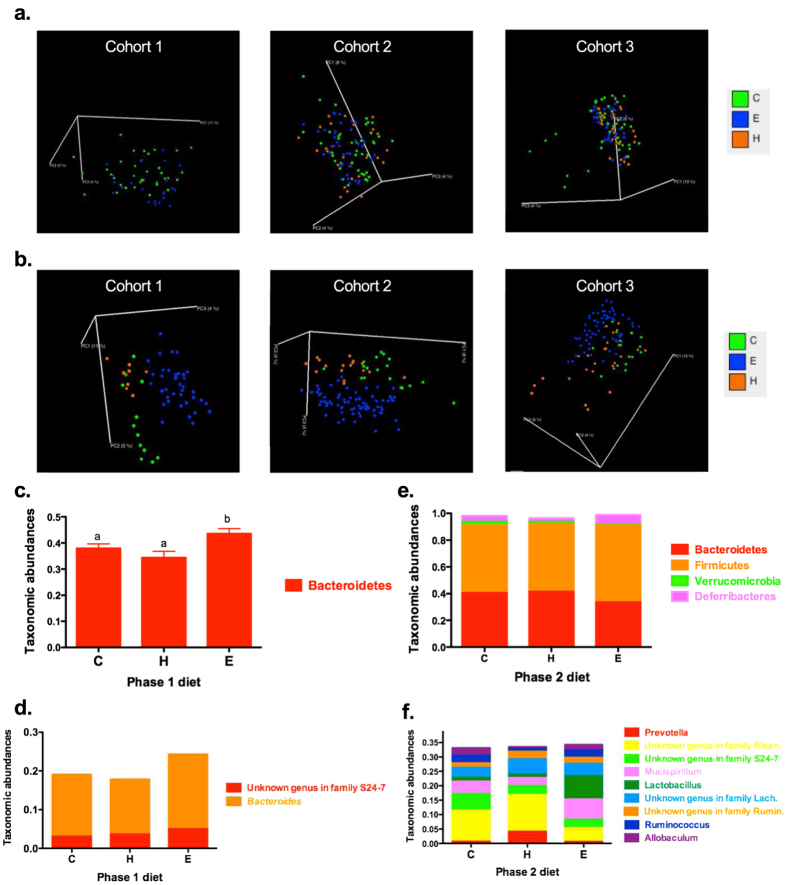
Colonic microbiota differs by diet. Diet interventions in phase 1 and phase 2 affected the microbiota structure in each individual cohort at the end of the study (60 weeks), showed in PCoA figures (**a**,**b**, respectively, not controlled for colonic sites). Each point corresponded to a sample colored by diet (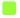: CON, 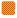: HF, and 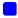: ER). Phase 1 diet significantly changed the relative abundances of one phylum (**c**) and two genera (**d**), while phase 2 diet significantly affected the relative abundances of four phyla (**e**) and nine genera (**f**). Different letters in the bar chart indicate significant different, p < 0.05.

In all three cohorts, diets during the initiation and progression phases were independent significant factors in modeling microbiota community structure (p = 0.006, p = 0.001, p = 0.003 for initiation phase diet, in three cohorts respectively; ps = 0.001 for progression phase diet in three cohorts; Fig. 3a,b). The clustering for initiation phase diets is less robust than those for progression phase diets in the principle coordinate analysis (PCoA) figures (Fig. 3a,b). At the phylum level, initiation phase diet only affected the relative abundance of Bacteroidetes (Fig. 3c). Animals on the ER diet during the initiation phase had a higher abundance of Bacteroidetes than those on the CON or HF diets (44%, 38%, 34%, respectively, ps < 0.05), regardless of their progression phase diet. At the genus level, after adjusting for cohort and sampling colonic location, the initiation phase diet had a long-term effect on the class Bacteroidia such that the abundances of genus *Bacteroides* and an unclassified genus in family S24-7 were higher in mice on the ER diet compared to mice on the CON or HF diets during this period (p < 0.005 and p < 0.0001, respectively; Fig. 3d).

Furthermore, the progression phase diet significantly changed the microbiota in four phyla and 9 genera. The ER diet in the progression phase reduced the relative abundances of Bacteroidetes compared to the HF diet (34%, 42%, respectively, p < 0.05, Fig. 3e). Animals on the ER diet in phase 2 also had higher relative abundances of Firmicutes and Deferribacteres (57%, 6.9%, respectively) than those on the HF diet (51%, 2.7%, respectively, ps < 0.05). The relative abundance of Verrucomicrobia did not differ between the ER diet and the HF diet in the progression phase, however, animals on the CON diet in the progression phase had a significantly higher abundance (0.8%, 1.5%, and 2.7%, respectively, ps < 0.001). The progression phase diet also affected the abundances of 9 genera with 3 from the phylum Bacteroidetes and 5 from the phylum Firmicutes after adjusting for the initiation phase diet, colonic location, and cohort effects (Fig. 3f). The abundances of bacteria in the genus *Prevotella,* an unclassified genus in *Lachnospiraceae,* and an unclassified genus in *Ruminococcaceae* were higher in mice on the HF diet in the progression phase compared to those on the CON or ER diet (p = 0.000002, p = 0.016, p = 0.0069, respectively). The adjusted abundances of genera *Ruminococcus* and *Allobaculum* were lower in mice on the HF compared to those on the other diets in phase 2 (p = 0.000001, p = 0.000004, respectively). The genera *Lactobacillus* and *Mucispirillum* were higher in mice on the ER diet compared to those on the CON or HF diets (p < 0.00001, p = 0.0015, respectively) in the progression phase. Unclassified genera in *Rikenellaceae* and *S24-7* were lower in mice on the ER diet compared to those on the CON or HF diets in the progression phase (p < 0.000001, p = 0.000004, respectively). When comparing across all 9 groups of dietary intervention, the relative abundances of 4 phyla and 13 genera were significantly changed by different combinations of dietary energy intake in early and adult life (means ± SE, Table 1). Genus *Prevotella* was significantly higher in the C-H group compared to others (7.4 ± 15.2%, p < 0.005). Genus *Ruminococcus* was significantly higher in the C-E, E-C, and H-E groups (2.8 ± 2.0%, 4.4 ± 3.9%, 3.8 ± 2.9%, respectively, p < 0.05). Genus *Bacteroides* showed the highest abundance in the E-E group and lowest in the H-H group (21.1 ± 12.7% and 9.5 ± 8.4%, respectively, p < 0.05).

### Colonic microbiota differs by colonic sites

**Figure 4:**
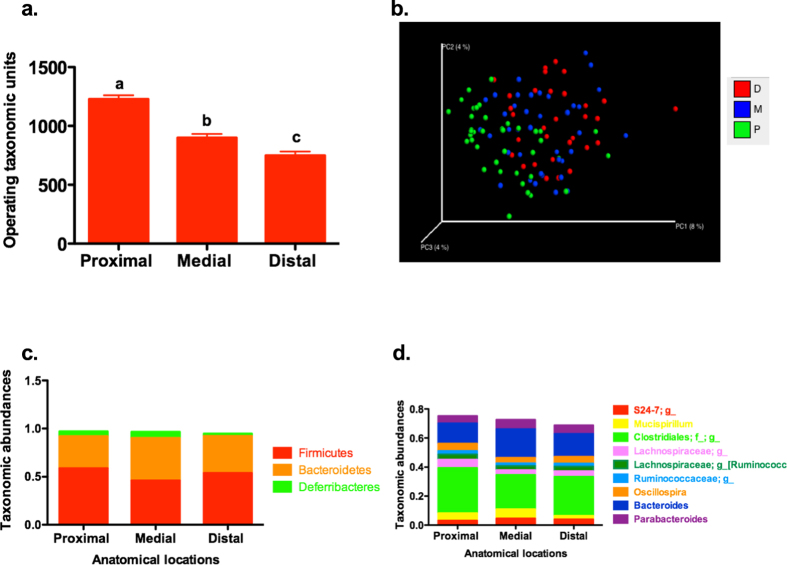
Microbiota differs by anatomical location in the colon. Significant differences by anatomical location were detected in OTU numbers (**a**). Beta diversity were significantly changed by location in three individual cohorts. The PCoA figure is presenting cohort 2 (**b**) (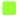: proximal colon, 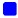: medial colon, 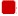: distal colon). Each point corresponded to a sample colored by colonic region (green: proximal; blue: medial; red: distal). Proportions of Bacteroidetes, Firmuctes, and Determibacteres were changed along the colon (**c**). Different letters in the bar chart indicate significant different, p < 0.05. OTU: Operational taxonomic units.

The overall microbiota varied by anatomical location in the colon (Fig. 4). Samples from the proximal colon had the highest OTU numbers compared to those from the medial or distal colon (Fig. 4a, p < 0.0001). Beta diversity measured by both unweighted (p = 0.001, R^2^ = 0.03798) and weighted UniFrac distances (p = 0.001, R^2^ = 0.06269) was significantly different by anatomical site, as demonstrated by PCoA (Fig. 4b, represented by cohort 2). The relative abundances of the three phyla were significantly changed by location in the colon (Fig. 4c). Firmicutes accounted for 59% of all sequences in the proximal colon, which was significantly higher than the medial colon (46%, p < 0.0001) or distal colon (53%, p < 0.05). The relative abundance of Bacteroidetes was lower (33%) in proximal colon compared to medial colon (44%, p < 0.0001). Deferribacteres accounted for 2.5% of all sequences in the distal colon, which was significantly lower than the medial colon (6.4%, p < 0.05), with proximal colon showing 5.1% (p = 0.269). A total of 9 genera were changed by colonic location (Fig. 4d). Five (5) genera in the phylum Firmicutes were higher in the proximal colon compared to the medial and distal sections (ps < 0.05), including two genera in the family *Lachnospiraceae* and two in the family *Ruminococcaceae.* Relative abundances of the genera *Mucispirillum, Bacteroides*, and *Parabacteroides* were higher in the medial section compared to the proximal and distal sections (ps < 0.05).

### Colonic microbiota differs by study cohort

**Figure 5:**
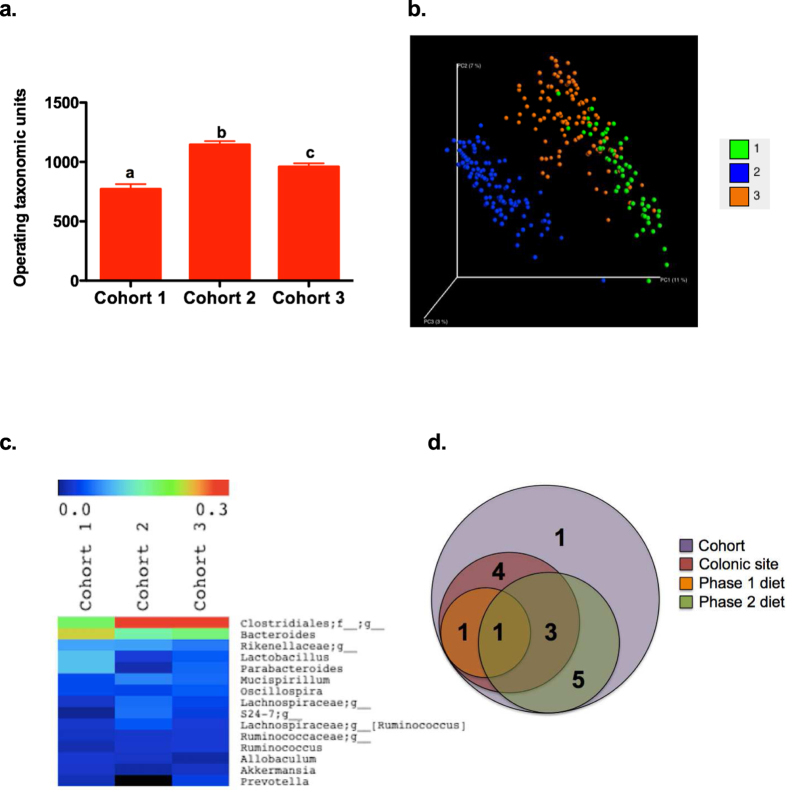
Microbiota differs by cohorts. OTU numbers of the colonic microbiota were significantly changed by cohorts, different letters indicate significant different, p < 0.05 (**a**). Variation in community diversity by cohort was shown in a PCoA figure (**b**). Each point represented a sample colored by cohort (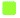: cohort 1; 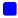: cohort 2; 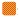: cohort 3). The significantly changed relative abundances of 15 genera by cohorts were shown in the heat map (**c**). Numbers in the Venn represented the number of genus significantly changed by phase 1 diet, phase 2 diet, anatomical locations in colon, and cohorts (**d**). Numbers in the overlapped area represented the number of same genus modified by multiple factors independently. OTU: Operational taxonomic units.

Although not a primary objective of the study, we chose to compare the three cohorts employed in the experiment to gain insight into issues that are very relevant to those pursuing similar studies where study animals may have a staggered start. The colonic microbiota was significantly different by cohort (Fig. 5). The OTU numbers from samples in cohort 2 were higher than those in cohort 1 and 3 (Fig. 5a, p < 0.0001). Initial analysis of Unifrac distance matrix in Adonis showed significant differences in microbiota across the three cohorts (unweighted p < 0.001, R^2^ = 0.03711; weighted p < 0.005, R^2^ = 0.02432). The PCoA figure furthered showed that cohort 2 clustered separately from cohorts 1 and 3 (Fig. 5b). Further investigation brought to attention that cohort 2 were from a different breeding facility than cohort 1 and 3. Moreover, relative abundances of 15 genera were significantly modified by cohort (Fig. 5c). Genera that were significantly changed by phase 1 diet, phase 2 diet, and colonic sites were nested in those 15 genera (Fig. 5d).

## Discussion

The number of studies regarding the impact of diet and GM will increase rapidly as the technology improves and the cost declines. It has been demonstrated that the healthy adult GM is characterized as existing in a steady state that requires a major disturbance, such as antibiotic administration or significant dietary intervention (magnitude or duration), to permanently alter that state. Both human and animal studies show an acute response of microbiota to dietary challenge^6^. However, it is unclear to what extent that energy balance over the life span influences the colonic microbiota after acute or chronic change. This raises a series of questions: (a) does early life dietary energy intake impose a community profile that remains stable in adulthood, regardless of diet change? (b) does a strong diet intervention (dramatic energy change and/or extended period of time) in adult life override the impact of early life dietary history? and (c) what are the health implications of the change in the GM resulting from alterations in energy balance? It is possible that different dietary interventions in early life shape the colonization and in turn influence the responses of microbiota to diet in adult life.

We designed a study to examine the impact of dietary energy variation prior to and during initiation versus progression in colon carcinogenesis in an AOM-induced colon cancer model. Unfortunately, the AOM dosing protocol previously described to induce colon cancer in FVB/N mice^24^ was not fully reproducible in our similar study and an insufficient number of colon cancers were detected to allow statistical analysis^25^. However, colonic ACFs, which is a precancerous colonic lesion and biomarker of cancer risk were carefully assessed^26^. We have demonstrated several novel findings regarding the colonic mucosal microbiota and colonic precancerous lesions in female mice: (a) ER diet during initiation phase enhances the carcinogenic impact of AOM while ER during progression inhibits the carcinogenesis cascade; (b) the impact of early dietary energy intake on the microbiota is partially retained into adulthood; (c) differences in dietary energy intake are reflected in the microbiota; (d) the microbiota changes from proximal to distal colon; and (e) the source of mice provided by the supplier, even within a given common strain, has an impact on the endogenous microbial population. These findings have significant implications for preclinical research and human studies.

Findings regarding body weight change in this study were as expected. In both phases, a HF diet with 45% kcal from fat increased body weight while the ER diet maintained the body weight at a significantly low level without symptoms of malnutrition. Consistent with previous studies, exposures to a HF diet in early life do not have a long-term effect on body weight in later life^27^. Moreover, animals on the ER diet in early phase of life gained weight after a switch to HF diet and have only moderately lower body weights (p>0.05) at the end of the study (60 weeks) compared to those on the HF or CON diet in early phase, suggesting that early-onset calorie restriction does not favor a lower body weight after a long period of time on HF diet in adult life. Similar to the effects on body weights, the ER diet during initiation had a lasting effect on body fatness after the same length of intervention of CON or HF diet during the progression phase. However, the effects were not permanent, overridden by the impact of diets during the longer progression phase, and disappeared at 60 weeks of age. It is clear that the duration of the change in energy balance and the magnitude of the change are both critical in their impact on body weight and adiposity, and a greater effort to obtain dose-dependent results will enhance our ability to translate these finding to human investigation.

The beneficial effects of ER diet during progression is consistent with previous studies, in which alterations in several biological pathways, including inflammation, have been proposed as possible mechanisms^16^. Retrospective studies demonstrate that energy restriction during childhood and adolescence reduces risk of colorectal cancer^28^. However, we found that prior to, and during initiation (AOM injections), an ER diet enhances development of subsequent precancerous lesions. The unexpected detrimental effects of early life ER diet may perhaps be explained by the altered AOM metabolism due to changes in body composition. Perhaps the pharmacokinetics of AOM is altered and in conjunction with the usual dosing on a total body weight basis, we have changed the activation or degradation of the carcinogen. During progression, HF diet enhanced ACF burden, which most certainly is mediated by different mechanisms^29^.

After controlling for cohort and colonic site impact by adding those factors in the statistical model, we observed significant effects of the diet on microbiota profiles. Although dietary energy intake in early life did not show a lingering impact on the number of OTUs, the impact on taxonomic abundance was observed in adulthood. Specifically, exposure to a HF diet in early life was associated with a significantly reduced proportion of Bacteroidetes in adult life, regardless of the dietary intervention in adulthood. The positive association between HF diet and reduced relative abundance of Bacteroidetes was consistent with a previous study^6^. Given the lack of longitudinal analysis of the microbiota at the end of the initiation phase compared to the end of the progression phase, we are unable to prove that the differentiated community structure was specifically retained from the earlier time point. However, it is clear that changes in energy balance early in life do have effects that are maintained for a long period of time in the mouse model. At genus level, the relative abundance of *Bacteroides* was significantly higher in animals on ER in phase 1 compared to those on HF. Certain species in *Bacteroides* have protective effects from inflammation in experimental models of inflammatory bowel disease (IBD)^30^, while some species induce colonic tumorigenesis in *Apc*^Min/+^ mice^31^. Proportion of *Bacteroides* also declined in animals challenged by psychological stressors^32^. The disagreement between the potential protective effects of increased *Bacteroides* and the rising number of ACF in mice on the ER diet during early life suggests that either bacteria species, which drive the increased proportion of *Bacteroides,* promoted colon carcinogenesis in the model, or the diet-associated *Bacteroides* abundance change is independent of observed diet-associated precancerous lesions alteration.

Most certainly the diet fed during the prolonged progression phase has a profound influences on the colonic microbiota. The progression diet did not change the number of OTUs, as noted for the diet during initiation. However, 4 phyla were significantly changed, regardless of past dietary energy intake history (initiation phase diet). Intriguingly, Bacteroidetes were higher in mice on the HF diet during the progression phase, compared to mice on the ER diet with the same dietary history (CON, HF, or ER in the initiation phase). However, we found that mice on the HF diet in both phases (H-H) still had lower proportion of Bacteroidetes compared to mice on the ER diet in both phases (E-E) (Table 1), which consistently demonstrated the association between HF diet and reduced abundance of Bacteroidetes. Moreover, the relative abundances of Bacteroidetes in mice on the ER diet in the initiation phase were higher than those on HF or CON in the initiation phase. Therefore, we hypothesized that the lower abundances of Bacteroidetes in animals on the ER diet during the progression phase may be largely due to mice fed the HF or CON diet during the initiation phase and the carry-on impacts of early life dietary patterns on Bacteroidetes abundances. On the other hand, the increased abundances of Firmicutes in the ER fed mice was likely due to the increased abundances of *Lactobacillus* (Table 1), which was previously reported to be inversely associated with social stress and inflammation^33^,^34^. ER diet has shown suppressive effects on colon carcinogenesis, which were associated with alterations in several biological pathways, including inflammation^16^. An ER diet has been shown to increase *Lactobacillus* group counts in a group of overweight adolescents^35^. Therefore, we hypothesized that the increased *Lactobacillus* by ER diet during the progression phase could be a mediator through which the suppressive effect of the ER diet can impact colon carcinogenesis. The differentiated effects of the ER diet on colonic microbiota by early life and adult life exposures indicated an interaction between the present colonic mucosal microbiota and the dietary intervention, further supporting the hypothesis that microbiota with similar structures may respond differently to specific dietary interventions while communities with different structures may respond differently to the same dietary interventions.

It is important for readers to understand that the dietary effects reported are in the context of the AOM-induced colon cancer model. This study was not designed to address the impact of the carcinogen on the microbiota structure and we do not have a parallel group of mice not exposed to AOM. However, one report indicates that 6 consecutive i.p. injections of AOM did not significantly impact on the GM composition or richness, even with cancer presented in the treatment group^36^. However, based upon the analysis we may not rule out the possibility that our results reflect an interaction between diet and AOM on the microbiota. Future studies are warranted and it is important to translate the current findings to studies addressing dietary changes on community structure in various models of colon carcinogenesis. In addition, we appreciate that although the 16S rRNA sequencing technique is a valuable tool to demonstrate relative changes in the community, the tool is limited in its ability to interpret the functional changes of the colonic mucosal microbiota.

We detected significant differences in microbiota profiles in different segments of the murine colon with adjustment for cohort and diet. In rodents treated with AOM, the microbiota differed by habitats along the gastrointestinal tract^37^. However, the regional variation in microbiota along the colon was scarcely reported in rodent models^38^. Human studies have not observed a quantitative or qualitative difference in bacteria from ileum to rectum^39^,^40^, although one study reported a positive trend between distances within the colon and quantitative differences^40^. The different OTU numbers, as well as taxonomic abundances at the genus level by colonic sites, observed in our study may be due to the fact that we examined samples from mice instead of human. The decreased diversity (measured by OTU numbers) in the distal colon could be explained by site-specific tumorigenesis in the AOM-induced colon cancer model. Although no gross tumors were observed along the colon in the mice that we sampled mucosal microbiota, nor did AOM change the luminal microbial composition in previous studies^36^, we cannot rule out the possibility that the decreased diversity and changed community structure between the anatomical locations along the colon were associated with morphological changes in the mucosa layer due to the toxigenic effect of AOM. The specific changes in taxonomic abundances along the colon we observed and the underlying mechanisms warrant further study. In addition, researchers should be aware of those changes in study design and data analysis.

As an unexpected observation, we found a significant cohort effect on the colonic microbiota. Indeed, study cohort independently changed all genera that were altered by either dietary intervention or anatomical location in the colon. Wild type C57BL/6N mice from the same company (Charles River, Wilmington, MA) are thought to be genetically identical. Considering that all three cohorts in this study were identically handled from week 3 to 60 regarding food, water, room temperature, and chemical intervention, it is possible that different maternal/environmental exposures in the first 2 or 3 weeks affected microbial colonization, which was retained over a year. Indeed, ordering records showed that the first and third cohorts were from the same distributor facility in Raleigh, NC, while the second cohort was from Kingston, NY, which aligned with the PCoA figure showing that cohort 2 clustered separately from cohort 1 and 3. Another breeding study observed similar results, reporting that the cohort effect accounted for 26% of the variation in the taxa of a core measureable microbiota defined by quantitative pyrosequencing^41^. The diversity of gut microbiota in pups decreased dramatically at 3 and 9 days after delivery in a mouse model^4^, suggesting that the microbiota in those days is more susceptible to environmental factors which might impact colonization. Therefore, the differences of microbiota profiles by cohorts in our study could be explained by environmental factors. Although interindividual variety has been largely addressed in previous studies, cohort effects were not widely appreciated. Our study here suggests that cohort effects should be controlled in gut microbiota-related animal studies.

Many investigators examining colon diseases in rodent models utilize pharmaceutical agents, dietary patterns, or specific nutrients or bioactive phytochemicals to assess impact on disease processes relevant to both prevention and therapy. Our work indicates that interventions altering dietary energy intake may act upon the colonic mucosal microbiota, changing both structure and possibly function, and thereby influencing biological or pathologic outcomes. Thus, energy intake, already known to be a powerful modulator of many host functions relevant to disease, is a crucial variable that should be addressed in all murine studies where microbiological outcomes are under investigation, as many interventions have an impact on food intake and growth. Furthermore, animal studies on gut microbiota typically utilize fecal samples considering cost and invasiveness relative to human studies. Our work implies that the impacts of cohort and sampling location should be considered, especially in research on the mechanistic role of gut microbiota in colon cancer, given that tumorigenesis may interplay with colonic mucosal microbiota and that tumors are more likely to develop in the distal colon in both humans and the AOM-induced colon cancer model. Finally, dietary intervention studies in humans targeting obesity and cancer prevention occasionally had null results due to residual confounders. Our work suggests that the lingering impacts of early life dietary history should be considered, particularly in studies where the microbes were proposed as a mediator, as the emerging role of gut microbiota in nutrition and health.

## Methods

### Study design

**Figure 6:**
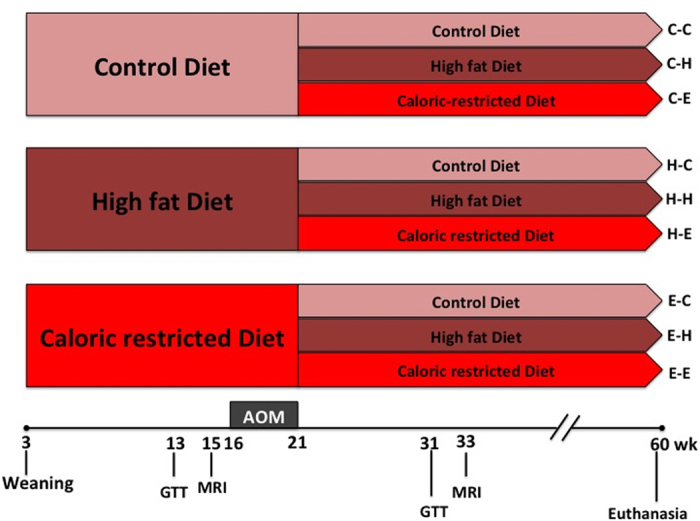
Study design. In phase 1 (3-to 21- weeks of age) and phase 2 (22-to 60- weeks of age) of the study, C57BL/6N female mice were fed control (CON, 10% kcal from fat), a high fat (HF, 45% kcal from fat), or a calorie restricted diet (ER, 30% caloric restricted compared to the control), leading to 3 × 3 groups of intervention at the endpoint (control- control (C-C), control-high fat (C-H), control-energy restriction (C-E), high fat-control (H-C), high fat- high fat (H-H), high fat-energy restriction (H-E), energy restriction-control (E-C), energy restriction-high fat (E-H), and energy restriction-energy restriction (E-E)). From 16- to 21- weeks of age, all mice received a weekly intraperitoneal injection of AOM at a dose of 10 mg/kg for 6 weeks. All mice were euthanized at 60 weeks of age and the colonic mucosal microbiota samples were collected in one representative animal in each cage at 60 weeks of age.

To model the impact of dietary patterns on the GM during the carcinogenesis process, we conducted a study where female C57BL/6N (Charles River, Wilmington, MA) mice were randomly assigned to either control, high fat or an energy restricted diet, as described below, starting at weaning (3 weeks) until 21 weeks of age. During weeks 16-21, animals received weekly AOM injections and therefore the phase 1 diets occurred prior to and during the initiation of the carcinogenesis process. Starting at week 22, until the end of the study at week 60 modeling the progression phase, animals were reassigned to one of the 3 diets. Thus, a total of 9 treatment groups were established as a 3 × 3 design (control- control (C-C, n = 27), control-high fat (C-H, n = 27), control-energy restriction (C-E, n = 27), high fat-control (H-C, n = 17), high fat- high fat (H-H, n = 13), high fat-energy restriction (H-E, n = 13), energy restriction-control (E-C, n = 20), energy restriction-high fat (E-H, n = 18), and energy restriction-energy restriction (E-E, n = 24)) (Fig. 6). At the end of 60 weeks animals were necropsied and colon prepared for ACF examination and colonic mucosal microbial analysis.

### Study Diets

Animals were assigned to one of three semi-purified diets (Research Diets, Inc. New Brunswick, NJ) (Table S1): 1) CON: a control diet (10% kcal from fat, D12450B); 2) HF: a high fat diet (45% kcal from fat, D12451); or 3) ER: a 30% calorie restricted diet (30% restricted compared to the control, D03020702).

### Azoxymethane induced colon carcinogenesis

At 16 weeks of age, all mice received a weekly intraperitoneal (i.p.) injection of azoxymethane (AOM) (Santa Cruz Biotechnology, Inc. Dallas, TX) at a dose of 10 mg/kg for 6 weeks. To manage the large sample size, the entire study was staggered and three identical cohorts of animals were obtained from Charles River (Wilmington, MA), each cohort has animals representing all nine combinations of dietary patterns. The entire experimental procedures were conducted according to the protocols approved by the Institutional Animal Care and Use Committee (IACUC) at The Ohio State University (protocol #2011A00000074).

### Mouse husbandry

All mice were housed in a barrier room in the animal facilities at The Ohio State University (OSU) with controlled temperature at 23 °C and a 12-h light/dark cycle. Mice (with dam) arriving at 2 weeks of age were acclimatized on CON diet for 1 week before weaning and initiating the study and had free access to water. Mice on CON or HF diet in either phase were group housed and fed *ad libtum*, while mice on ER diet in either phase were individually housed and fed daily to ensure equal dietary energy intake. Standard approved enrichments were added to each cage to minimize environmental stress.

### Body measurements

Body weight was measured individually every week. Body fat was measured in a randomly selected cohort of 4–9 mice per diet group at 15, 33, and 60 weeks of age via magnetic resonance imaging (MRI) in the Small Animal Imaging Core (SAIC) at the Ohio State University. Briefly, the mouse body was segmented from the background using Otsu segmentation. A connected components algorithm was used to label the background objects in the image and ‘fill’ any holes in the segmented body image. A global threshold of 120 grey level intensity was chosen to segment fat from surrounding tissue. The whole body and segmented fat mask was then used to calculate the percentage of fat in the whole body.

### Colon tissue harvest and ACF quantification

At week 60, colonic mucosal tissues from one representative animal in each cage (n = 1 ~ 9 per group in each cohort) were harvested for analysis to minimize cage effects on the microbiota. Before euthanasia, all animals were fasted for 10–12 hours. During necropsy, the colon was excised under aseptic conditions and gently rinsed in sterile cold phosphate buffered saline (PBS) (Fisher Scientific, PA). The entire colon was divided into three segments of equal length (proximal, medial, and distal). A 3 mm piece of tissue of each section was harvested and collected in sterile tubes for bacterial analyses. Tissue was frozen in liquid nitrogen and stored at −80 °C for analysis. An approximately 15 mm segement of each section was fixed in 10% neutral buffered formalin and stained with methylene blue to identify ACF under microscope. Total number of ACF in all three sections was recorded for each animal.

### Bacterial analyses

DNA Isolation and Sequencing: DNA isolation and paired-end 2 × 300 Illumina MiSeq sequencing was performed by Molecular Research LP MR. DNA (Shallowater, TX). DNA was isolated from proximal, distal, and medial sections of colon tissues using the MoBIO PowerSoil kit (Valencia, CA). The V1-V3 16s rRNA gene primers 27F/534r were used in a 30 cycle PCR with the HotStarTaq Plus Master Mix (Qiagen, Carlsbad, CA). The thermoprofile used was: 1 cycle at 94 °C for 3 mins, 28 cycles at 94 °C for 30 s, 53 °C for 40 s and 72 °C for 60 s, ending with an elongation step of 72 °C for 5 mins. Amplicon quality was verified via agarose gel, then pooled and purified with Ampure XP beads. The Illumina TruSeq DNA library preparation protocol was used for library prep, then amplicons were loaded for sequencing in the Illumina MiSeq machine, following the manufacturer’s guidelines. MR. DNA filtering of final sequences included: removal of sequences <150 base-pairs long and removal of sequences with ambiguous base-calls. Samples were trimmed based upon a quality score of 25 and then joined. Final .fasta and .qual files were provided to the investigators.

Sequence Analysis: Fasta and .qual files and were filtered in QIIME (version 1.8.0)^42^ based upon: 200-650bp in length, maximum ambiguous bases set at 6, minimum qual score of 25, max. homopolymer run of 6, and zero allowed primer mismatches. 95.15% of sequences passed filtering, resulting in 65,294 sequences/samples (300 total samples). Operational taxonomic units (OTUs) picking was performed using tool set qiime-tools (http://github.com/smdabdoub/qiime-tools), a method that makes use of parallel BLAST OTU picking^43^,^44^. In summary, qiime_tools splits .fasta files into smaller datasets for rapid picking using the Ohio Supercomputer with the parallel_blast_pick_otus.py command. OTUs were picked against the GreenGenes 13_8 97% OTUs reference database^45^. After the resulting OTU results files were merged into one overall table, taxonomy was assigned based upon the gg_13_8 reference taxonomy.

### Statistical analysis

Body weights (gm), body fatness (%), and total number of ACF were reported as means ± SEM and analyzed using a two-way analysis of variance (ANOVA) controlled for cohort effect. To determine which groups were significantly different from one another, Bonferroni post hoc tests were conducted in SAS (version 9.3, Cary, NC).

Microbial sequence data were pooled for OTUs comparison and taxonomic abundance analysis but separated by batch in principle coordinates analysis (PCoA) to have clear PCoA figures. For even sampling, a depth of 10,000 sequences/sample was used. PCoAs were produced using Emperor^46^. Community diversity was determined by the number of OTUs and beta diversity, measured by UniFrac unweighted and weighted distance matrices in QIIME^47^. Microbiota composition analysis was determined by taxonomic abundances which were limited to genera with at least 1% abundance. The abundances were normalized by finding square root of proportion, then the arcsine of the square root.

Adonis^48^, a permutational multivariate analysis of variance, was used to determine statistically significant clustering of groups based upon microbiota structure distances. Due to large impacts of cohort on the community structure, the impacts of phase 1 diet and phase 2 diet were analyzed on each individual cohort. ANOVA for unbalanced sample with multiple factors, including phase 1 diet, phase 2 diet, cohort, and colonic segment, was performed on taxonomic abundances with Bonferroni correction in SAS to determine significant effects of those factors (version 9.3, Cary, NC). Significance was set at p < 0.05.

## Additional Information

**How to cite this article**: Xu, J. *et al.* The Impact of Dietary Energy Intake Early in Life on the Colonic Microbiota of Adult Mice. *Sci. Rep.* **6**, 19083; doi: 10.1038/srep19083 (2016).

## Supplementary Material

Supplementary Information

## Figures and Tables

**Table 1:** Relative abundances of phyla and genera changed by diet intervention (%, means ± SEM).

Phase 1 diet (wks 3 to 21)	Control (C)			High-fat (H)			Energy restriction (E)			*p*
Phase 2 diet (wks 22 to 60)	C	H	E	C	H	E	C	H	E	
Bacteroidetes	43.7 ± 3.6^a^	43.4 ± 3.5^a^	30.9 ± 2.0^a^	29.4 ± 5.7^a^	32.7 ± 4.9^a^	32.3 ± 2.9^a^	44.4 ± 4.1^a^	45.9 ± 4.7^a^	39.0 ± 2.0^a^	0.0007
Bacteroidia										
*Prevotella*	1.2 ± 1.0^a^	7 ± 0.9^b^	0.5 ± 0.6^a^	0.2 ± 1.6^a^	0.5 ± 1.3^a^	0.6 ± 0.8^a^	0.8 ± 1.1^a^	3 ± 1.3^ab^	1.1 ± 0.6^a^	<0.0001
Unknown genus in Rikenellaceae	14 ± 1.1^a^	13 ± 1.0^a^	4.1 ± 0.6^b^	5.9 ± 1.7^bc^	12 ± 1.5^ac^	5 ± 0.9^b^	10 ± 1.2^ac^	13 ± 1.4^ac^	6 ± 0.6^b^	<0.0001
Unknown genus in S24-7	4.5 ± 0.7^ac^	1.3 ± 0.7^a^	2.6 ± 0.4^ab^	3.9 ± 1.1^abd^	3.8 ± 0.9^bc^	2.8 ± 0.5^abc^	8.1 ± 0.8^d^	5.5 ± 0.9^cd^	3.5 ± 0.4^bc^	<0.0001
*Bacteroides*	16 ± 2.3^ab^	16 ± 2.2^ab^	17 ± 1.3^ab^	15 ± 3.7^ab^	12 ± 3.2^b^	16 ± 1.9^ab^	18 ± 2.7^ab^	15 ± 3.1^ab^	22 ± 1.3^a^	0.0107
*Parabacteroides*	7.2 ± 0.8^ab^	5.3 ± 0.7^b^	5.1 ± 0.4^ab^	3.7 ± 1.2^ab^	4.2 ± 1.0^ab^	7.1 ± 0.6^a^	6.1 ± 0.9^ab^	6.9 ± 1.0^ab^	5.9 ± 0.4^ab^	0.0021
Deferribacteres	4.5 ± 2.2^ab^	1.7 ± 2.1^b^	8.8 ± 1.2^a^	6.2 ± 3.5^ab^	3.7 ± 3.0^ab^	8.3 ± 1.8^a^	2.8 ± 2.5^b^	4.4 ± 2.9^ab^	3.8 ± 1.3^ab^	0.0031
Deferribacteres										
*Mucispirillum*	4.5 ± 2.2^ab^	1.7 ± 2.1^b^	8.8 ± 1.2^a^	6.2 ± 3.5^ab^	3.7 ± 3.0^ab^	8.3 ± 1.8^a^	2.8 ± 2.5^b^	4.4 ± 2.9^ab^	3.8 ± 1.3^ab^	0.0031
Firmicutes	47.7 ± 3.6^a^	46.3 ± 3.5^a^	58.0 ± 2.0^a^	61.1 ± 5.8^a^	62.2 ± 5.0^a^	56.7 ± 2.9^a^	46.4 ± 4.2^a^	46.8 ± 4.8^a^	55.4 ± 2.1^a^	0.0034
Bacilli										
*Lactobacillus*	0.4 ± 1.6^b^	0.3 ± 1.5^b^	7.6 ± 0.9^a^	2.2 ± 2.5^b^	2.1 ± 2.2^b^	8 ± 1.3^a^	2.1 ± 1.8^b^	1.7 ± 2.1^b^	8.7 ± 0.9^a^	<0.0001
Clostridia										
Unknown genus in Clostridiales	25 ± 2.9^abc^	23 ± 2.8^c^	28 ± 1.6^abc^	41 ± 4.6^a^	38 ± 4.0^ab^	25 ± 2.3^abc^	20 ± 3.3^bc^	26 ± 3.8^abc^	25 ± 1.6^abc^	0.0022
Unknown genus in Lachnospiraceae	3.4 ± 0.8^ab^	4.8 ± 0.7^ab^	3.9 ± 0.4^ab^	5.3 ± 1.2^ab^	6.4 ± 1.1^a^	4.1 ± 0.6^ab^	1.8 ± 0.9^b^	4.9 ± 1.0^ab^	4.1 ± 0.4^ab^	<0.0190
Unknown genus in Ruminococcaceae	1.8 ± 0.3^a^	3.0 ± 0.3^a^	2.3 ± 0.2^a^	1.2 ± 0.5^a^	2.8 ± 0.4^a^	2.3 ± 0.3^a^	1.5 ± 0.4^a^	1.7 ± 0.4^a^	2.1 ± 0.2^a^	<0.0449
*Ruminococcus*	2.3 ± 0.4^abc^	1.2 ± 0.4^b^	2.8 ± 0.2^ac^	1.0 ± 0.7^bc^	0.9 ± 0.6^b^	3.4 ± 0.3^a^	4.2 ± 0.5^a^	0.9 ± 0.5^b^	1.7 ± 0.2^b^	<0.0001
Erysipelotrichi										
*Allobaculum*	2.1 ± 0.8^ac^	0.6 ± 0.7^b^	1.4 ± 0.4^abc^	1.0 ± 1.2^abc^	0.3 ± 1.1^b^	0.5 ± 1.0^abc^	3.8 ± 0.9^a^	0.5 ± 1.0^bc^	2.3 ± 0.4^abc^	<0.0001
Verrucomicrobia	2.4 ± 0.5^a^	2.0 ± 0.5^ab^	0.6 ± 0.3^b^	1.9 ± 0.8^ab^	0.4 ± 0.7^b^	1.3 ± 0.4^ab^	3.4 ± 0.6^a^	1.5 ± 0.6^ab^	0.9 ± 0.3^b^	<0.0001
Verrucomicrobiae										
*Akkermansia*	2.4 ± 0.5^a^	2.0 ± 0.5^ab^	0.6 ± 0.3^b^	1.9 ± 0.8^ab^	0.4 ± 0.7^b^	1.3 ± 0.4^ab^	3.4 ± 0.6^a^	1.5 ± 0.6^ab^	0.9 ± 0.3^b^	<0.0001

Different letters denote significant differences in pair-wise comparisons (p < 0.05). *p-*value in ANOVA for unbalanced sample with Bonferroni adjustment.
