# Supplementary material for: The Impact of Dietary Energy Intake Early in Life on the Colonic Microbiota of Adult Mice
Source: Sci Rep. 2016 Jan 8;6:19083. doi: 10.1038/srep19083 (PMC4705468; doi:10.1038/srep19083)
Supplement: Supplementary Information [file srep19083-s1.pdf]

The Impact of Dietary Energy Intake Early in Life on the Colonic Microbiota of Adult Mice

Jinyu Xu[1,2], Jeffrey D. Galley[3], Michael T. Bailey[3,4,5,6], Jennifer M. Thomas-Ahner[4], Steven K. Clinton[4,7], and Susan E. Olivo-Marston[2,4,*]

[1] The Ohio State University Ph.D. Program in Nutrition, The Ohio State University, Columbus, OH 43210, USA
[2] Division of Epidemiology, College of Public Health, The Ohio State University, Columbus, OH 43210, USA
[3] Division of Biosciences, College of Dentistry, The Ohio State University, Columbus, OH 43210, USA
[4] Comprehensive Cancer Center, The Ohio State University, Columbus, OH 43210, USA
[5] Institute for Behavioral Medicine Research, The Ohio State University Wexner Medical Center, Columbus, OH 43210, USA
[6] Center for Microbial Pathogenesis, The Research Institute at Nationwide Children's Hospital, Columbus OH 43205, USA
[7] Division of Medical Oncology, Department of Internal Medicine, The Ohio State University, Columbus, OH 43210, USA
* Author to whom correspondence should be addressed. olivo-marston.1@osu.edu

Product	CON		HF		ER	
	gm	kcal	gm	kcal	gm	kcal
Protein	19.2%	20%	23.7%	20%	27.0%	29%
Carbohydrate	67.3%	70%	41.4%	35%	54.0%	57%
Fat	4.3%	10%	23.6%	45%	6.0%	14%
Kcal/gm	*3.85*		*4.73*		*3.78*	
**Ingredient (per 1000 g)**						
Casein, 80 Mesh	200	800	200	800	200	800
L-Cystine	3	12	3	12	3	12
Corn Starch	315	1 260	72.8	291	197.9	792
Maltodextrin 10	35	140	100	400	0	0
Sucrose	350	1 400	172.8	691	197.9	792
Cellulose, BW200	50	0	50	0	50	0
Soybean Oil	25	225	25	225	25	225
Lard	20	180	177.5	1 598	20	180
Mineral Mix S10026	10	0	10	0	10	0
DiCalcium Phosphate	13	0	13	0	13	0
Calcium Carbonate	5.5	0	5.5	0	5.5	0
Potassium Citrate, 1 H2O	16.5	0	16.5	0	16.5	0
Vitamin Mix V10001	10	40	10	40	10	40
Choline Bitartrate	2	0	2	0	2	0

Supplementary Table 1. Diet composition and calorie distribution. The formula[1] to calculate the exact amount of food for mice on ER diet in either phase was provided by the company (Research Diets, Inc. New Brunswick, NJ). To ensure 30% energy restriction is equal for all animals on ER in phase 2, reference animals were the CC (control-control) group.

[1] *The amount of ER diet for each animal/day = 0.71 × the average amount of CON diet consumed by the reference animals in the previous day.*

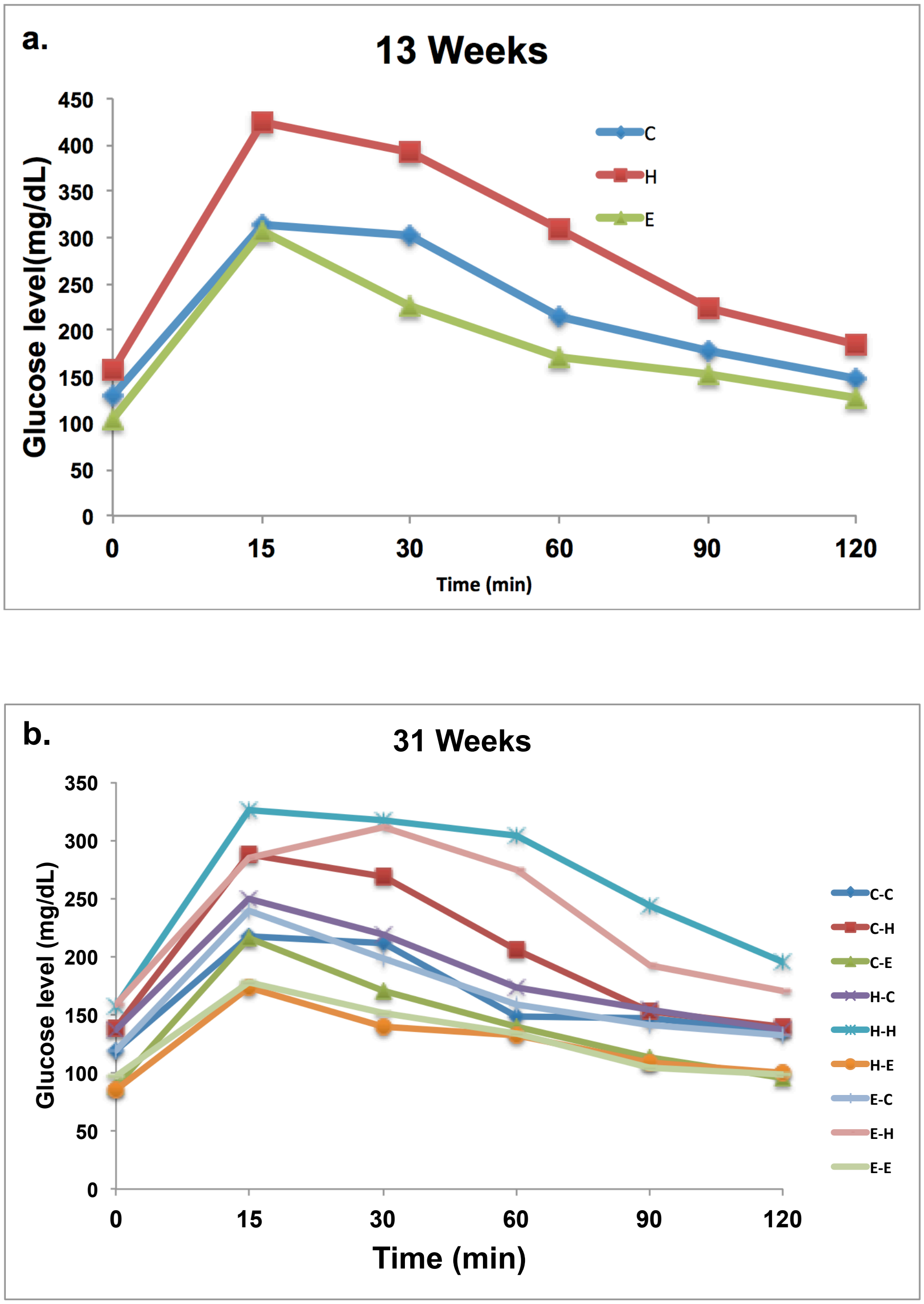

Supplementary Figure 1. Blood glucose levels during glucose tolerance testing. Response to glucose tolerance testing at 13 (a) and 31 (b) weeks of age are shown (n=10). Diet patterns are represented by letter codes C (10% kcal from fat, control diet), H (45% kcal from fat, high fat diet), and E (30% caloric restricted compare to control, caloric restricted diet). Each data point represents the mean blood glucose level per dietary group.

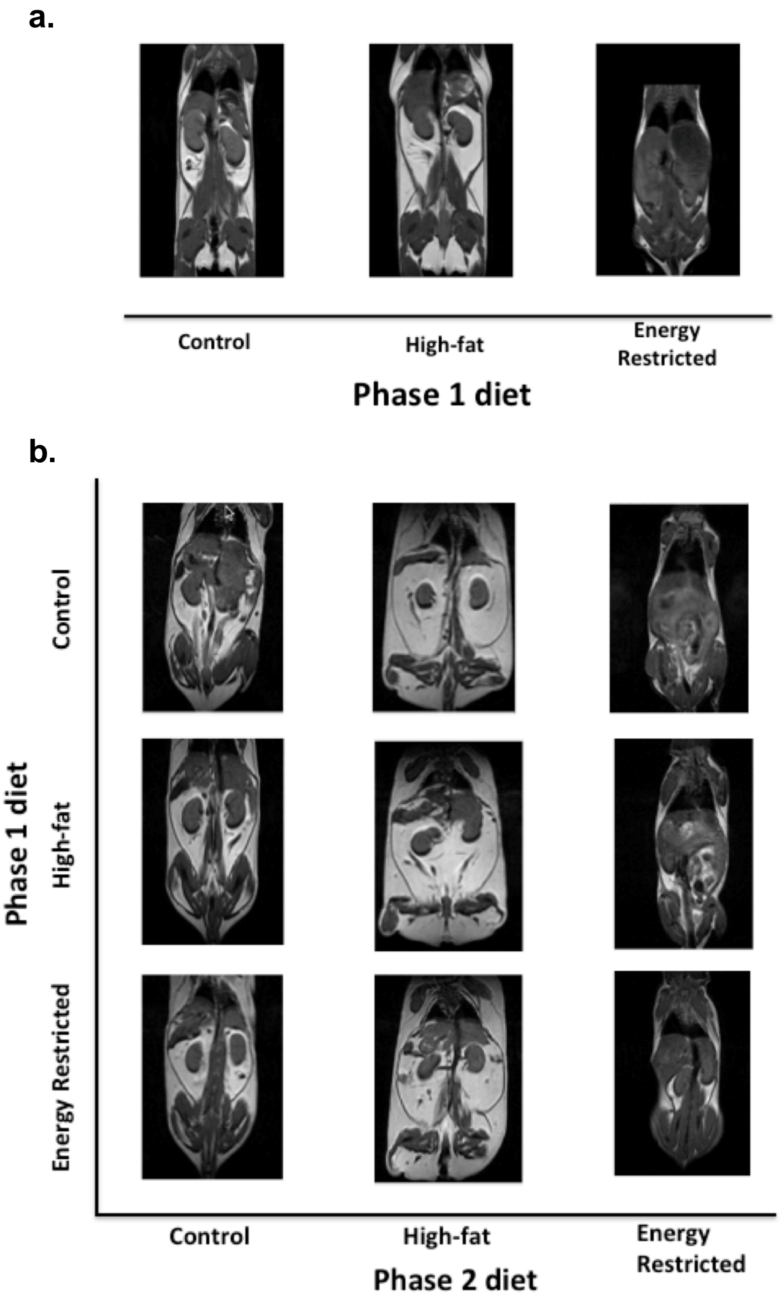

Supplementary Figure 2. Magnetic resonance imaging (MRI) showing body fat distribution. Body fat at 15 (a) and 60 (b) weeks of age are shown by MRI. Bright area indicates fat tissue.
